# Awareness, Attitudes, and Behavioral Practices of the Population of the Republic of Kazakhstan Regarding Tuberculosis

**DOI:** 10.3390/healthcare14060790

**Published:** 2026-03-20

**Authors:** Nadira Aitambayeva, Altyn Aringazina, Temur Yeshmuratov, Laila Nazarova, Bekdaulet Akimniyazova, Tatyana Popova, Sholpan Aliyeva, Akmaral Savkhatova, Nazerke Narymbayeva, Shnara Svetlanova, Akylbek Saktapov

**Affiliations:** 1 Department of Public Health and Social Sciences, Kazakhstan Medical University “KSPH”, Almaty 050060, Kazakhstan; 2 School of Health Sciences, Almaty Management University (AlmaU), Almaty 050060, Kazakhstan; altyn.aringazina@gmail.com; 3 Department of Surgery, Kazakh-Russian Medical University, Almaty 050000, Kazakhstan; 4 Department of Epidemiology EBM and Biostatistics, Kazakhstan Medical University “KSPH”, Almaty 050060, Kazakhstan; zauirbekovna92@gmail.com; 5 Department of Pulmonology, S.D. Asfendiyarov Kazakh National Medical University, Almaty 050012, Kazakhstan; 6 Gynecological Department, Hospital of the Medical Center of the Presidential Administration, Almaty 050000, Kazakhstan; 7 Radiotherapy Department, Kazakh Institute of Oncology and Radiology, Almaty 050060, Kazakhstan; 8 Department of Healthcare Management, Kazakhstan Medical University “KSPH”, Almaty 050060, Kazakhstan; n.narymbay@gmail.com; 9 Department of Nursing, Kazakhstan Medical University “KSPH”, Almaty 050060, Kazakhstan; 10 Department of Epidemiology, Biostatistics and Evidence-Based Medicine, Al-Farabi Kazakh National University, Almaty 050040, Kazakhstan

**Keywords:** tuberculosis, people living with HIV (PLHIV), injecting drug users (IDUs), internal migrants, risk factors, tuberculosis prevention, access to health services, social determinants of health, Kazakhstan

## Abstract

**Highlights:**

**What are the main findings?**

- Awareness of tuberculosis remains low among the general population in Kazakhstan, particularly among internal migrants.
- People living with HIV and individuals who use drugs demonstrate higher knowledge of TB symptoms and testing; however, stigma and misconceptions persist across all groups.

**What is the implication of the main finding?**

- The findings indicate a critical need for targeted tuberculosis awareness campaigns tailored to vulnerable populations, especially internal migrants. Strengthening training programs for healthcare workers and implementing inclusive, culturally sensitive communication strategies may improve public understanding of tuberculosis, reduce stigma, and enhance early detection and treatment outcomes. These measures are essential for improving TB prevention and control in Kazakhstan.

**Abstract:**

Background: This study aims to examine the level of awareness, attitudes (including stigma and discrimination), and behaviors related to tuberculosis among the population of the Republic of Kazakhstan to identify priorities for raising awareness and reducing stigma. Methods: The study interviewed 2400 people from six regions of Kazakhstan using stratified random sampling based on gender and age. Respondents were chosen from cities and villages, including RK citizens over 18 who could answer questions. Additionally, 400 people with HIV, 200 drug users, 200 internal migrants, and 500 health workers were interviewed. Recruitment was done through profile organizations and the snowball method, with all participants giving informed consent. Results: The study showed different levels of knowledge about tuberculosis (TB) in Kazakhstan. Radiography was the most commonly known detection method (71–91%). Awareness of sputum testing was highest among drug users (84%) and HIV patients (77%), but lower among internal migrants (39%). Internal migrants had the most uncertainty about TB tests (17%). Stigmatizing views of TB patients existed, with 28–38% believing most people reject them. Among healthcare workers, only 38. 8% correctly identified the G-Xpert test for TB and rifampicin resistance, and over one-third misunderstood the Mantoux test’s purpose. Conclusions: The findings show a need for focused educational efforts to boost TB awareness and lessen stigma, especially among internal migrants and the general public. Vulnerable groups, like PLHIV and PWUD, have higher awareness but still encounter major barriers. Improving healthcare workers’ knowledge about TB diagnostics is also crucial. Specific communication strategies and policies are needed to improve TB detection, reduce social stigma, and improve healthcare access for at-risk groups in Kazakhstan.

## 1. Introduction

According to the National Center for AIDS Prevention and Control of the Republic of Kazakhstan, tuberculosis is diagnosed in 45.8% of patients with HIV infection at the symptomatic stage. It is the cause of death in approximately 36% of people living with HIV [1]. Despite improvements in the epidemiological situation for tuberculosis (TB), Kazakhstan still faces challenges, including an increase in drug-resistant forms of the disease, the detection of advanced forms, and patient interruptions of treatment. An analysis of the epidemiological situation for tuberculosis (TB) in the regions of the Republic of Kazakhstan from 2018 to 2024 revealed a steady decline in TB incidence, while mortality risks remained, particularly in the pre-pandemic and COVID-19 pandemic periods. Before the pandemic, the incidence rate reached 48.2 cases per 100,000 population, and the mortality rate was 2.4 cases. By 2024, these figures had decreased to 33.5 (−30.5%) and 1.0 (−58.3%), respectively, reflecting improvements in the post-pandemic period. The highest concentrations of TB cases were observed in the northern, western, and southern regions of the country, with higher incidence rates persisting in regions with high unemployment after the pandemic. Adults remained the most vulnerable group, and the lack of a consistent correlation between vaccination rates and incidence confirms the multifactorial nature of TB spread in Kazakhstan [2,3]. Reducing HIV-related stigma in healthcare settings should remain a priority in Kazakhstan, and the need to simultaneously address the stigma associated with injection drug use is also highlighted. Assessing health care workers’ attitudes and behaviors and identifying key intervention points is the first step toward effectively addressing this issue. Stigma is reinforced by social norms, low awareness of anti-discrimination policies, and weak enforcement. People living with HIV and people who inject drugs face denial of services, negative attitudes, segregation, and non-consensual disclosure of their HIV status [4]. Knowledge levels varied significantly across populations and geographic locations. In Kazakhstan, people living with HIV and people who use drugs demonstrated higher awareness of the severity of tuberculosis (77% and 86%, respectively) compared to internal migrants (64%) and the general population (69%). Similarly, these vulnerable groups were more likely to report airborne transmission (94%) compared to the general population (79%) and internal migrants (78%). Several studies have documented low overall knowledge levels despite high general awareness. In Jordan, although 90.1% of participants had heard of tuberculosis, 88.9% of Jordanians, 92.8% of urban refugees, 92% of camp refugees, and 90.5% of migrants had low levels of knowledge about tuberculosis. The study found low levels of knowledge about tuberculosis and high levels of stigma among target groups, highlighting the need for health education programs on the causes, symptoms, and modes of transmission of TB, as well as on combating stigma, especially among vulnerable groups in Jordan [5]. A study conducted in The Gambia found that while 83.9% of participants had heard of tuberculosis, only 66.9% had sufficient knowledge about it. In India, only 17% of respondents had adequate knowledge of tuberculosis [6]. Gaps in knowledge regarding the causes and mechanisms of tuberculosis transmission were particularly noticeable. In Ethiopia, only 25.8% knew that tuberculosis was caused by bacteria, despite 95.5% having heard of the disease. A study among Ethiopian pastoralists found that 63.9% of pastoralists cited bacteria as the cause of the disease, compared to 81% among the sedentary population, and that 53.6% of pastoralists associated tuberculosis with witchcraft, compared to 23.5% among the sedentary population. In Pakistan, only a quarter of respondents mentioned airborne transmission, and 18% were unaware of the mode of transmission [7]. Awareness of tuberculosis symptoms was mixed. In Inner Mongolia, only 26.9% knew that a cough lasting three weeks or more was a sign of tuberculosis [8]. In Pakistan, only 7% knew that a cough lasting 2–3 weeks was a classic symptom. However, in The Gambia, symptom awareness was relatively high, with persistent cough, weight loss, and chest pain frequently reported [9]. In Thailand, a study of 3074 general population individuals and 559 family members of TB patients found that migrants and ethnic minorities had lower levels of TB knowledge than the general population, as measured by mean knowledge scores in a multi-stage, stratified cross-sectional sample [10]. In Tajikistan, a study of 509 migrant workers revealed increased vulnerability to tuberculosis due to working conditions abroad and limited access to healthcare due to their legal status and the cost of treatment. The average level of knowledge about TB was low, and misconceptions were widespread. Despite free anti-TB drugs in the country, diagnosis and supportive treatment are paid for, creating a financial burden. Improving early diagnosis and accessible treatment for migrants both abroad and at home is a priority [11]. Tuberculosis remains the leading cause of death in low- and middle-income countries. A review of 30 studies found low levels of knowledge about TB, high stigma, and limited access to health care, especially among the poorly educated and socially vulnerable groups. Educational programs, stigma-reduction measures, and innovative solutions to improve diagnosis and treatment are needed [12]. Additional challenges in providing medical care are observed among key populations. Studying the level of tuberculosis awareness among the general population and specific vulnerable groups will help improve information strategies to foster appropriate public attitudes and behaviors toward tuberculosis and reduce stigma and discrimination.

## 2. Materials and Methods

### 2.1. Study Design and Population

This study is a one-stage sociological survey using questionnaires and in-depth interviewing. In-depth interviews were part of the broader project and were used to provide contextual insight; the present manuscript, however, focuses exclusively on the survey-based quantitative component.

### 2.2. Sample Size and Sampling Method

No gender distribution was assumed in the planned study. The study included participants of both sexes without quantitative differentiation. There was no distribution based on nationality. The study included participants of any nationality without restrictions. The study was conducted in 6 regions of Kazakhstan: one large city and 2 villages were selected from each region.

### 2.3. Process of Tool Development

The survey instrument is based on the example of the questionnaire recommended by WHO in the guidelines for the design of knowledge, attitude, and practice studies [13]. Questionnaires were developed separately for each group of respondents and presented in Kazakh and Russian. These included a questionnaire for the general population; a questionnaire for PLHIV; a questionnaire for PWUD; a questionnaire for internal migrants; a questionnaire for medical workers; and an in-depth interview program. Informed consent was obtained from each respondent before the study began. The questionnaires were administered on paper and completed by the respondents themselves. At the same time, respondents from vulnerable groups were remunerated for participation in the interview. The completed questionnaires were collected and handed over to the customer after data processing and analysis. Formal pilot testing was not conducted; the questionnaires were reviewed by public health experts to ensure clarity and relevance of the items.

### 2.4. Data Collection

**Figure 1:**
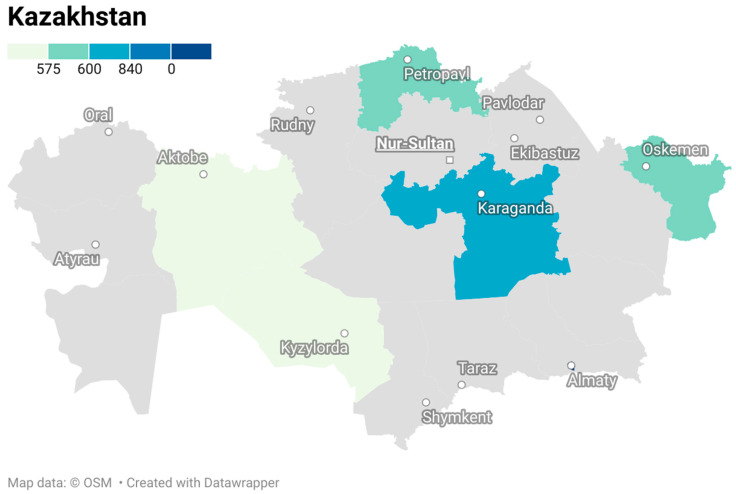
Number of respondents living with HIV, people who use drugs and health workers by region of Kazakhstan.

The total number of respondents included 3700 people: general population; people living with HIV (PLHIV); people who use drugs (PWUD); internal migrants (IM); healthcare workers (HCWs). The distribution of study participants is presented in Figure 1.

General population. Total sample size—2400 people: in Almaty city and Almaty region—400 people, in Kyzylorda region400 people, in North-Kazakhstan region—400 people, in Aktobe region—400 people, in East-Kazakhstan region—400 people, in Karaganda region—400 people.

Inclusion criteria: (1) Age over 18 years; (2) citizens of the Republic of Kazakhstan.

Exclusion criteria: (1) Under 18 years of age; (2) non-residents of the Republic of Kazakhstan; (3) persons unable to adequately answer the questions due to various circumstances.

Search and recruitment of respondents from the general population. A combined approach was used to form the sample: stratification and random sampling. The selection of routes and respondents at the survey points was carried out as follows:

Selection of routes. In cities, the procedure for selecting routes was as follows: streets for the survey were selected at random, with equal representation of different city districts. The selection of the starting house on the route was done using a random number generator—each interviewer was given information about the selected route for the survey—the street and the serial number of the house from which to start the survey. The interviewer had to start moving along the route from the indicated house number. In case the selected house number did not exist or there was an administrative or non-residential building under this number, the interviewer was directed to the nearest, next house number. Then, the interview was conducted in each subsequent house—in a plot with apartment buildings, and in every 3rd house—in plots of individual construction. In the case where the number of flats in a house is between 4 and 12, the survey was carried out in every 3rd house. The initial direction of movement along the street is in the direction of increasing house numbers on one side of the street (even or odd side). After half of the respondents of the route assignment have been interviewed or in the case when the street is over—the interviewer proceeds in the opposite direction on the other side of the street. Administrative, industrial buildings, as well as all types of dormitories, were not included in the sample; the interviewer skipped these buildings.

In rural settlements, the survey streets were selected as follows: if the village has a conventional division into parts or districts, the survey was conducted on the streets in each part. If there is no division of the village, the main street of the village and one or 2–3 perpendicular streets were selected for the survey. Or if there are no perpendicular streets, the next parallel street is selected. In rural settlements, the survey was conducted in every 3rd house of an individual building. The initial direction of movement along the street was toward increasing house numbers on the odd side, and after half of the respondents were interviewed, or if the street ended, in the opposite direction on the even side of the street.

Flat selection. Apartment selection was carried out in each house allocated for the survey by systematic sampling. In houses with the number of flats 4–12, the selection of flats was carried out randomly. In each of the following houses, the number of flats in which the survey was conducted was excluded from the selection. If the flat turned out to be uninhabited (rented out as an office, tenants moved out, etc.), the interviewer did not find anyone at home, or received a refusal, he moved on to the neighboring flat with a higher number.

The selection of a respondent in the household was carried out according to a quota by sex and age. If there were several eligible respondents, the selection was based on the principle of ‘last birthday’. Only household members permanently residing in the flat participated in the survey.

A total of 400 people living with HIV were interviewed. In three oblasts: North-Kazakhstan, East-Kazakhstan and Karaganda oblasts 70 people living with HIV were interviewed each. In Kyzylorda and Aktobe oblasts, 50 people were interviewed each. In Almaty, 90 people with HIV were interviewed. Access to this social group is organized with the help of NGOs working with people living with HIV, regional/city AIDS prevention and control centers.

Inclusion criteria: (1) age over 18 years; (2) men and women with confirmed status of living with HIV; (3) urban and rural residents.

Exclusion criteria: (1) under 18 years of age; (2) persons unable to adequately answer the questions due to various circumstances.

Finding and recruiting respondents. Letters were sent to the regional/city AIDS prevention and control centers (AIDS Centers) to obtain permission to conduct the study at the centers. Then, infectious disease doctors randomly selected PLHIV from the total number of PLHIV and invited them to participate in the study on predetermined days at the (AIDS Centers) base. To ensure confidentiality and ethical standards, the survey was conducted by interviewers who did not have access to lists and personal data of respondents. The questionnaires were used only if the respondent signed a paper version of informed consent.

People who use drugs (PWUD). Total sample—200 people: in Kyzylorda, North-Kazakhstan, Aktobe and East-Kazakhstan oblasts—25 people each, in Karaganda oblast and Almaty city—50 people each. Access to people who use drugs will be organized with the help of Mental Health Centers.

Inclusion criteria: (1) age over 18 years; (2) male and female drug users; (3) urban and rural residents.

Exclusion criteria: (1) under 18 years of age; (2) persons unable to adequately answer the questions due to various circumstances.

Finding and recruiting respondents. Letters were sent to regional/city mental health centers to obtain support for the study. The specialists of the mental health center randomly selected drug users from the general database and invited them to participate in the study at a specified time. The survey was conducted by interviewers who did not have access to the lists of drug users and personal data of the respondents to ensure confidentiality and ethical standards. The questionnaires were used only if the respondent signed a paper-based informed consent.

Internal migrants. A total of 200 people were interviewed among internal migrants. The survey was conducted in Almaty city and Almaty oblast as the region with the largest number of internal migrants. In this study, an internal migrant is a person who has moved within the last year within the country for permanent or temporary earnings.

Inclusion criteria: (1) age over 18 years; (2) men and women who have left/entered Almaty and Almaty region for work in the last year.

Exclusion criteria: (1) age below 18 years; (2) men and women who have not left/entered Almaty for work in the last year; (3) persons unable to adequately answer the questions due to various circumstances.

Search and recruitment of respondents. This category of respondents was recruited in places where internal migrants congregate to look for work: job search ‘spots’, construction and non-food markets, construction sites, agricultural fields, while providing services to private individuals (repair, construction, agricultural work, private carriage/taxi services, etc.). The method of searching for this category is ‘snowballing’. The survey was conducted by ethical interviewers after respondents signed informed consent.

Medical workers. A total of 500 medical workers (general practitioners and district nurses) were interviewed: in Kyzylorda, North-Kazakhstan, Aktobe, East-Kazakhstan, Karaganda oblasts—80 people each, in Almaty city—100 people. To ensure equal probability sampling, urban primary health care (PHC) medical organizations (private and public) were randomly selected from the current lists of medical organizations. For this purpose, the approved lists of medical organizations in each oblast covered by the Mandatory Social Health Insurance (MSHI) were used. In each oblast, 8–13 medical organizations were covered.

Finding and recruiting respondents. Lists of general practitioners and district nurses were requested from each organization selected for the survey, and then the required number of respondents was randomly selected. Four general practitioners and four district nurses were interviewed from each health organization included in the study. The survey was conducted at the allotted time by interviewers after obtaining informed consent from the respondents.

Descriptive and comparative statistics were used to analyze the data. Quantitative variables were summarized using mean ± standard deviation, median, and interquartile range. The Kolmogorov–Smirnov test was used to assess normality. Since the data were not normally distributed, nonparametric tests were used for comparisons between groups: the Kruskal–Wallis test for multilevel comparisons and the Mann–Whitney test for paired comparisons. Categorical variables were analyzed using the χ^2^ (chi-square) test and Fisher’s exact test for small sample sizes. All statistical calculations were performed in SPSS version 25.0, with a significance level of 0.05.

Differences in awareness of TB detection methods between the general population and internal migrants (χ^2^ = X, *p* < 0.05).

Differences in attitudes toward TB patients between PLHIV and PWUD (χ^2^ = Y, *p* < 0.05).

## 3. Results

### Socio-Demographic Characteristics of Survey Participants

A total of 2405 respondents participated in the survey, exceeding the planned sample size of 2400 by five respondents, all from Almaty. Among them, 200 were internal migrants, 400 were people living with HIV (PLHIV), and 200 were people who use drugs (PWUD). The sample included representatives of the general population, internal migrants, people living with HIV (PLHIV), and people who use drugs (PWUD). Socio-demographic characteristics are summarized in Table 1. Statistically significant differences were identified between groups in gender, age, education level, and marital status (X^2^ test, *p* ≤ 0.05 for all comparisons).

According to the data, males accounted for 48–76% across subgroups, while females accounted for 23% to 52%. Respondents ranged from under 30 to over 50 years; the largest proportion of PLHIV and PWUD were in the 31–50 age range. Most respondents had secondary or higher education. Internal migrants had higher proportions of incomplete secondary education. Furthermore, between 24 and 62% of respondents were married. Internal migrants represented the highest proportion of never-married individuals, 33%. Most respondents preferred Russian (50–90%), except internal migrants, where 50% preferred Kazakh.

The average duration of stay in Almaty for internal migrants was 4.5 months (range 1–10 months). Among PLHIV, the average duration since HIV diagnosis was 6.8 years (range 1 month–38 years). PWUD reported an average duration of drug use of 14.4 years (range 0.5–45 years).

Radiography was the most frequently identified method for tuberculosis detection, with recognition rates ranging from 71% to 91% as shown in Table 2. Internal migrants demonstrated the lowest awareness at 71% and exhibited the highest proportion of “don’t know” responses at 24%. Additional methods identified included the Mantoux test (12–29%), microscopy (6–21%), and molecular genetic methods such as G-Xpert (3–11%). Comparative analysis indicated that people living with HIV and people who use drugs (PWUD) exhibited significantly higher awareness of radiography as a detection method compared to internal migrants (X^2^ = 45.3, *p* ≤ 0.001). Awareness of the Mantoux test also varied significantly among these groups (X^2^ = 32.1, *p* ≤ 0.001).

There were significant differences in knowledge of diagnostic tests among individuals with suspected tuberculosis (Table 3). Awareness of sputum testing was higher among people who use drugs (84%) and people living with HIV (77%) compared to internal migrants (39%) (Manna-Whitney U test, *p* ≤ 0.001). Awareness of blood testing ranged from 28% to 35% across groups, with significant differences observed (X^2^ = 18.5, *p* = 0.002). The proportion of “don’t know” responses was higher among internal migrants (17%) compared to other groups (3% to 7%) (X^2^ = 56.2, *p* ≤ 0.001). Sputum testing was correctly identified by 54% of the general population, 39% of migrants, 77% of PLHIV, and 84% of PWUD. Blood tests were noted by 28–35% of respondents. Internal migrants more frequently responded “don’t know” (17%) compared to other groups (3–7%).

Community attitudes demonstrated ongoing stigma, as shown in Table 4. Between 32% and 38% of respondents believed that most people reject individuals with tuberculosis (TB). Between 44% and 64% indicated that people are friendly toward TB patients but tend to avoid them. Supportive attitudes were reported by only 5% to 18% of respondents. Internal migrants reported the highest proportion of friendly but avoidant attitudes (64%), which was significantly higher than the general population (48%) and people living with HIV (51%) (X^2^ = 26.4, *p* ≤ 0.001).

**Figure 2:**
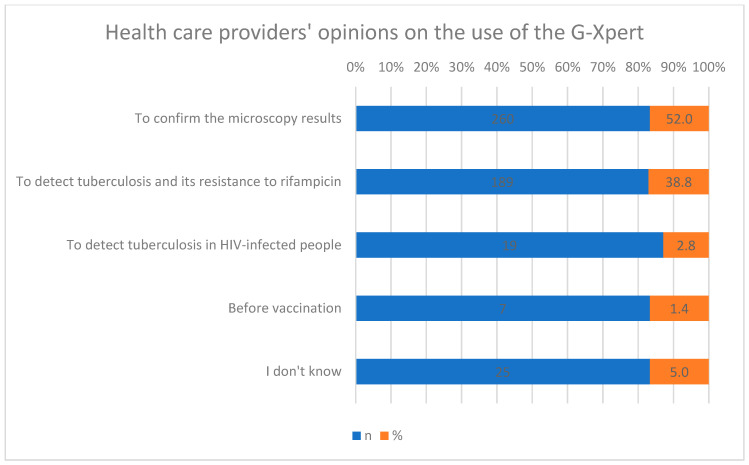
Health professionals’ views on the use of the G-Xpert assay.

Regarding the G-Xpert assay, 52% of health workers indicated it is necessary to confirm microscopy results; 38.8% identified its role in detecting tuberculosis and rifampicin resistance, 2.8% cited its use in pre-vaccination testing for HIV-infected children, and 5% reported uncertainty. For Mantoux testing, 58.4% of participants reported its use in screening children, 37% for diagnosis, 3.6% for vaccination, 0.8% for treatment monitoring, and 0.2% did not respond. Significant differences in knowledge regarding the purposes of Mantoux testing were observed among health workers (X^2^, *p* ≤ 0.001) (Figure 2).

**Figure 3:**
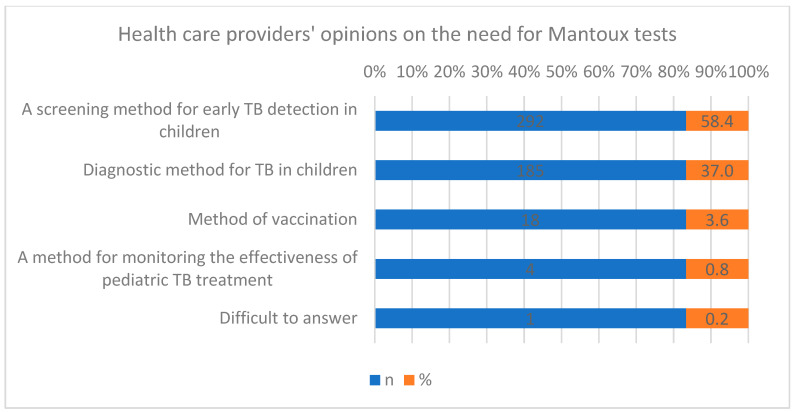
Health workers’ views on the need for a Mantoux test.

A survey on the purpose of Mantoux testing showed that 58.4% of health workers consider Mantoux as a screening method for early detection of TB in children, while 37.0% of respondents consider Mantoux as a method for diagnosing TB in children, 3.6% as a method of vaccination, 0.8% as a method for monitoring the effectiveness of TB treatment in children, and 0.2% of health workers found it difficult to answer (Figure 3).

## 4. Discussion

This study identified low levels of tuberculosis awareness among the population of Kazakhstan, with levels varying by socio-demographic and clinical characteristics. Internal migrants exhibited particularly limited knowledge, as evidenced by the highest proportion of “don’t know” responses and the lowest awareness of diagnostic methods, including radiography, the Mantoux test, and molecular genetic testing (G-Xpert). The association between lower knowledge levels and more negative social attitudes among internal migrants is derived from group-level comparisons. No analysis at the individual level was performed to examine this relationship. These findings indicate the presence of structural and social barriers, such as restricted access to medical information, language preferences, and residential instability, which align with international research on migrants and other vulnerable groups. A systematic review demonstrated that the incidence and prevalence of tuberculosis among refugees and migrants are substantially higher than those in host countries. These results underscore the need to enhance tuberculosis prevention and control efforts among vulnerable populations [14]. These conditions create barriers to timely diagnosis and treatment, which may contribute to delays in detection and spread of infection [15]. Concurrently, individuals living with HIV and those who inject drugs exhibited greater knowledge of tuberculosis symptoms and diagnostic methods. This increased awareness is likely attributable to their more frequent engagement with specialized medical and social services, which offer access to educational programs and informational resources [16,17,18]. However, even among these groups, stigmatizing attitudes persist, highlighting the need to integrate educational and anti-stigmatizing interventions into prevention strategies.

The study’s findings also revealed a link between knowledge about tuberculosis and the level of stigma. Groups with lower awareness were more likely to demonstrate negative attitudes toward tuberculosis patients, which is consistent with theoretical models showing that a lack of information increases fear and prejudice [19].

Individuals living with HIV and those who inject drugs demonstrated greater knowledge of tuberculosis (TB) symptoms and diagnostic methods, likely due to their regular engagement with health and social services. However, misconceptions and stigma remain prevalent, even within these populations. These findings underscore the need for comprehensive educational initiatives and stigma-reduction efforts. Significant knowledge gaps were also identified among primary care providers, including general practitioners and district nurses. Many were unfamiliar with contemporary diagnostic tools such as GeneXpert and the Mantoux test, which may hinder early detection and referral. Only 38.8% of specialists accurately identified the purpose of GeneXpert, while many either responded incorrectly or expressed uncertainty. These results highlight the need for targeted training for general practitioners and nurses to enhance their proficiency with current TB diagnostic methods. The analysis further revealed differences between specialist types and regions, indicating that training should be adapted to local contexts. Regarding social attitudes, most respondents reported avoiding or subtly discriminating against TB patients, with supportive attitudes being uncommon. Internal migrants were more likely to report friendly yet avoidant attitudes (64%), a pattern associated with limited awareness and illustrating the relationship between knowledge and stigma. The findings show that vulnerable groups need targeted information and education strategies. Internal migrants and people with lower levels of education benefit from multilingual communication and clear information about symptoms and diagnoses. People living with HIV and those who inject drugs need ongoing programs that help with early detection and sticking to treatment [20]. Furthermore, the high level of awareness of radiography among respondents is consistent with the widespread practice of fluorographic and radiographic screening in Kazakhstan. However, the low level of knowledge about sputum bacteriological testing and molecular genetic diagnostic methods (including GeneXpert) reflects a gap between modern diagnostic algorithms recommended by national protocols and the level of awareness among the general public and some healthcare workers.

### Strengths and Limitations

A major strength of this study is its comprehensive and representative sample, which included both the general population and key vulnerable groups (PLHIV, PWUD, internal migrants, and healthcare workers) across multiple regions of Kazakhstan. The use of stratified random sampling and validated WHO-recommended tools ensured methodological rigor and data reliability. Additionally, the use of both quantitative surveys and in-depth interviews allowed for a richer understanding of TB awareness and stigma. While the general population survey is designed to be representative at the level of the surveyed regions, the recruitment of vulnerable groups through social service networks and snowball sampling may limit the generalizability of these findings beyond the included participants and settings.

However, the study also has several limitations. First, self-reported data may be subject to response bias, particularly on sensitive topics such as drug use or HIV status. Second, while the snowball sampling method facilitated access to hard-to-reach populations, it may have introduced selection bias. Third, the cross-sectional nature of the study limits causal inferences. Finally, language and literacy barriers may have influenced responses, despite the availability of surveys in both Kazakh and Russian. Another limitation is that formal pilot testing of the questionnaires was not conducted prior to the survey. However, the instrument was reviewed by public health experts to ensure clarity and contextual relevance.

## 5. Conclusions

Tuberculosis awareness in Kazakhstan is notably low, especially among internal migrants who lack knowledge of symptoms and diagnostics. Despite higher awareness in people living with HIV and drug users, stigma and misconceptions persist across all groups, including healthcare workers. Targeted educational programs should focus on migrants and healthcare providers, emphasizing symptom recognition and modern diagnostics. Culturally adapted communication and anti-stigma efforts are crucial. These findings highlight gaps in TB awareness, urging improved public education and professional training for better prevention and treatment.

Communication strategies for internal migrants should be multilingual and oriented toward symptom recognition and timely utilization of key diagnostic tests. Training programs for primary healthcare staff should focus on practical interpretation and application of GeneXpert and Mantoux tests, addressing the documented knowledge gaps. These targeted recommendations directly respond to the specific gaps identified in the study, enhancing their relevance for policy and practice.

## Figures and Tables

**Table 1:** Socio-demographic characteristics of population and internal migrant respondents, people living with HIV (PLHIV) and people who use drugs (PWUD).

Features		Population (*n* = 1405)		Internal Migrants (*n* = 200)		People Living with HIV (*n* = 400)		People Who Use Drugs (PWUD) (*n* = 200)		*p*-Value
		*n*	%	*n*	%	*n*	%	*n*	%	
Gender	Male	1165	48.4%	115	57.5%	245	61.3%	153	76.5%	*p* ≤ 0.001
	Female	1240	51.6%	85	42.5%	155	38.8%	47	23.5%	
Age group	30 years and under	773	32.1%	65	32.5%	89	22.3%	34	17.0%	*p* ≤ 0.001
	31–40	594	24.7%	75	37.5%	161	40.3%	64	32.0%	
	41–50	482	20.0%	44	22.0%	120	30.0%	71	35.5%	
	More than 50	556	23.1%	16	8.0%	30	7.5%	31	15.5%	
Education level	Primary education (grades 3–4)	9	0.4%	2	1.0%	1	0.3%	3	1.5%	*p* ≤ 0.001
	Incomplete secondary (8–9 grades)	66	2.7%	39	19.5%	44	11.0%	52	2.0%	
	Secondary general (10–11 grades)	419	17.4%	98	49.0%	132	33,0%	67	33.5%	
	Secondary school	936	38.9%	46	23.0%	166	41.5%	61	30.5%	
	Higher education	888	36.9%	15	7.5%	54	13.5%	17	8.5%	
	Post-education (master’s degree)	80	3.3%	-	-	3	0.8%	-	-	
	Post-education (doctoral studies)	7	0.3%	-	-	-	-	-	-	
Marital status	Never married	577	24.0%	67	33.5%	124	31.0%	53	26.5%	*p* ≤ 0.001
	Married	1489	62.0%	100	50.0%	166	41.5%	79	39.5%	
	Divorced	226	9.4%	26	13.0%	94	23.5%	61	30.5%	
	Widows	113	4.7%	7	3.5%	16	4.0%	7	3.5%	
Preferred language for obtaining information	Kazakh	887	36.9%	99	49.5%	47	11.8%	19	9.5%	
	Russian	1518	63.1%	101	50.5%	353	88.3%	181	90.5%	

Note: “-” indicates that no participants were observed in this category.

**Table 2:** Awareness of TB detection methods.

Total	Population		Internal Migrants		People Living with HIV		People Who Use Drugs (PWUD)		*p*-Value
	N	%	*n*	%	N	%	*n*	%	
	2405	100.0%	200	100.0%	400	100.0%	200	100.0%	
Radiography	2039	85.0%	141	71.0%	362	91.0%	176	88.0%	*p* ≤ 0.001
Microscopy	340	14.0%	12	6.0%	85	21.0%	23	12.0%	*p* ≤ 0.001
Molecular genetic method—G-Hregt	134	6.0%	6	3.0%	45	11.0%	19	10.0%	*p* ≤ 0.001
Bronchoscopy	329	14.0%	16	8.0%	56	14.0%	32	16.0%	0.02
Enzyme-linked immunosorbent assay (ELISA) analysis	101	4.0%	2	1.0%	39	10.0%	25	13.0%	*p* ≤ 0.001
Mantoux test	707	29.0%	23	12.0%	101	25.0%	50	25.0%	*p* ≤ 0.001
I don’t know	149	6.0%	48	24.0%	17	4.0%	9	5.0%	*p* ≤ 0.001
Sputum analysis	-	-	-	-	6	2.0%	9	5.0%	*p* ≤ 0.001

Note: “-” indicates that no participants were observed in this category.

**Table 3:** Awareness of tests to be used in suspected tuberculosis cases.

Total	Population		Internal Migrants		People Living with HIV		People Who Use Drugs		*p*-Value
	*n*	%	*n*	%	*n*	%	*n*	%	
	2405	100.0%	200	100.0%	400	100.0%	200	100.0%	
Blood	1105	46.0%	65	33.0%	141	35.0%	56	28.0%	0.002
Phlegm	1289	54.0%	77	39.0%	306	77.0%	167	84.0%	*p* ≤ 0.001
Saliva	322	13.0%	48	24.0%	38	10.0%	6	3.0%	*p* ≤ 0.001
Pharyngeal swab	93	4.0%	4	2.0%	9	2.0%	7	4.0%	0.21
I don’t know	176	7.0%	34	17.0%	12	3.0%	6	3.0%	*p* ≤ 0.001
Urine	4	0.2%	1	1.0%	2	1.0%	-	-	0.05
All kinds of analyses	4	0.2%	6	3.0%	-	-	-	-	0.01
Fluorography	2	0.1%	-	-	-	-	-	-	0.99

Note: “-” indicates that no participants were observed in this category.

**Table 4:** Attitudes of different population groups towards people with tuberculosis.

Total	Population		Internal Migrants		People Living with HIV		People Who Use Drugs		*p*-Value
	N	%	*n*	%	N	%	*n*	%	
	2405	100%	200	100%	400	100%	200	100%	
Most people reject him or her	767	32.0%	56	28.0%	133	33.0%	75	38.0%	0.01
Most people are friendly, but they usually try to avoid him or her	1163	48.0%	128	64.0%	203	51.0%	88	44.0%	*p* ≤ 0.001
The community is mostly supportive and helpful to him or her	414	17.0%	10	5.0%	56	14.0%	36	18.0%	*p* ≤ 0.001
Indifferently	13	1.0%	-	-	-	-	-	-	
No discrimination	15	1.0%	-	-	2	0.5%	-	-	0.12
Fear of such	3	0.1%	-	-	-	-	-	-	
Negative	2	0.1%	-	-	-	-	1	0.5%	
Refusal to answer	28	1.2%	-	-	-	-	-	-	
Difficult to answer	-	-	6	3.0%	6	1.5%	-	-	

Note: “-” indicates that no participants were observed in this category.

## Data Availability

All data generated or analyzed during this study are included in this article. Further inquiries can be directed at the corresponding author.

## Abbreviations

The following abbreviations are used in this manuscript:

TB	Tuberculosis
HIV	Human Immunodeficiency Virus
DR-TB	drug-resistant tuberculosis
WHOQOL-HIV BREF tool	World Health Organization Quality of Life

## References

[1] Juszkiewicz K., Jarosz M., Włoszczak-Szubzda A., Głowacka M. (2020). Effectiveness of Tuberculosis Prophylaxis in Patients with HIV/AIDS—Retrospective Analysis of Data from Almaty, Kazakhstan, 2010–2015. Ann. Agric. Environ. Med..

[2] Bekshin Z., Askarov A., Abduraimov Y., Rsaliyev A., Bissenova G., Amirkhanova N., Nurbekova Z., Temirbekova A. (2025). Tuberculosis and Impact of COVID-19 on Spread of Epidemics in Kazakhstan. Pathogens.

[3] Turgenbayev K.A., Borsynbayeva A.M., Plazun A.A., Turgenbayev R.K. (2021). Tuberculosis Prevalence in Animals and Humans in the Republic of Kazakhstan. Vet. World.

[4] Rees N., Tod D., Fiorentino F., O’Meara P., Williams L., Williams J., Hawkes C. (2025). Attitudes towards Protecting Emergency Medical Services (EMS) Staff from Violence and Aggression: A Survey of Adults in Wales. BMJ Open.

[5] Alsoukhni M.A., Khader Y., Abaza H., Wilson N., Satyanarayana S. (2023). Tuberculosis-Related Knowledge, Behaviors, Stigmatizing Attitude, and Discrimination among Refugees, Migrants, and the General Population in Jordan. SAGE Open Med..

[6] Sagili K.D., Satyanarayana S., Chadha S.S. (2016). Is Knowledge Regarding Tuberculosis Associated with Stigmatising and Discriminating Attitudes of General Population towards Tuberculosis Patients? Findings from a Community Based Survey in 30 Districts of India. PLoS ONE.

[7] Khan A., Shaikh B.T., Baig M.A. (2020). Knowledge, Awareness, and Health-Seeking Behaviour Regarding Tuberculosis in a Rural District of Khyber Pakhtunkhwa, Pakistan. BioMed Res. Int..

[8] Ma E., Ren L., Wang W., Takahashi H., Wagatsuma Y., Ren Y., Gao F., Gao F., Wang W., Bi L. (2015). Demographic and Socioeconomic Disparity in Knowledge About Tuberculosis in Inner Mongolia, China. J. Epidemiol..

[9] Bashorun A.O., Linda C., Omoleke S., Kendall L., Donkor S.D., Kinteh M.-A., Danso B., Leigh L., Kandeh S., D’Alessandro U. (2020). Knowledge, Attitude and Practice towards Tuberculosis in Gambia: A Nation-Wide Cross-Sectional Survey. BMC Public Health.

[10] Pengpid S., Peltzer K., Puckpinyo A., Tiraphat S., Viripiromgool S., Apidechkul T., Sathirapanya C., Leethongdee S., Chompikul J., Mongkolchati A. (2016). Knowledge, Attitudes, and Practices about Tuberculosis and Choice of Communication Channels in Thailand. J. Infect. Dev. Ctries..

[11] Gilpin C., De Colombani P., Hasanova S., Sirodjiddinova U. (2011). Exploring TB-Related Knowledge, Attitude, Behaviour, and Practice among Migrant Workers in Tajikistan. Tuberc. Res. Treat..

[12] Craciun O.M., Torres M.D.R., Llanes A.B., Romay-Barja M. (2023). Tuberculosis Knowledge, Attitudes, and Practice in Middle- and Low-Income Countries: A Systematic Review. J. Trop. Med..

[13] World Health Organization (2008). Advocacy, Communication and Social Mobilization for TB Control: A Guide to Developing Knowledge, Attitude and Practice Surveys.

[14] Wong C.S., Chidgey A., Lee K.L., Mo P.K.H., Wong T., Banerjee S., Ho V., Leow Y., Gowindah R., Yew Y.J. (2024). Empowering People Living with HIV (PLHIV): Unveiling Care Gaps and Identifying Opportunities for Improving Care for PLHIV in Singapore and Hong Kong. J. Int. AIDS Soc..

[15] Sahan S., Topluoglu S., Temel F., Gokler M.E., Kaygusuz S. (2023). Did Financial Social Support for Tuberculosis Patients Lead to Better Treatment Outcomes in Türkiye during 2018–2019?. Jpn. J. Infect. Dis..

[16] Hermosilla S., El-Bassel N., Aifah A., Terlikbayeva A., Zhumadilov Z., Berikkhanova K., Darisheva M., Gilbert L., Schluger N., Galea S. (2015). Tuberculosis Report among Injection Drug Users and Their Partners in Kazakhstan. Public Health.

[17] Kidder D.P., Fierro L.A., Luna E., Salvaggio H., McWhorter A., Bowen S.-A., Murphy-Hoefer R., Thigpen S., Alexander D., Armstead T.L. (2024). CDC Program Evaluation Framework, 2024. Recomm. Rep..

[18] Degenhardt L., Webb P., Colledge-Frisby S., Ireland J., Wheeler A., Ottaviano S., Willing A., Kairouz A., Cunningham E.B., Hajarizadeh B. (2023). Epidemiology of Injecting Drug Use, Prevalence of Injecting-Related Harm, and Exposure to Behavioural and Environmental Risks among People Who Inject Drugs: A Systematic Review. Lancet Glob. Health.

[19] Pourcher V., Gourmelen J., Bureau I., Bouee S. (2020). Comorbidities in People Living with HIV: An Epidemiologic and Economic Analysis Using a Claims Database in France. PLoS ONE.

[20] Wu S., Litvinjenko S., Magwood O., Wei X. (2023). Defining Tuberculosis Vulnerability Based on an Adapted Social Determinants of Health Framework: A narrative review. Glob. Public Health.

